# Impact of clinical registries on quality of patient care and clinical outcomes: A systematic review

**DOI:** 10.1371/journal.pone.0183667

**Published:** 2017-09-08

**Authors:** Dewan Md Emdadul Hoque, Varuni Kumari, Masuma Hoque, Rasa Ruseckaite, Lorena Romero, Sue M. Evans

**Affiliations:** 1 Department of Epidemiology and Preventive Medicine, School of Public Health and Preventive Medicine, Monash University, The Alfred Centre, Melbourne, Victoria, Australia; 2 Maternal and Child Health Division, International Centre for Diarrhoeal Diseases Research in Bangladesh, Dhaka, Bangladesh; 3 The Ian Potter Library, The Alfred Centre, Melbourne, Victoria, Australia; University of South Australia, AUSTRALIA

## Abstract

**Background:**

Clinical quality registries (CQRs) are playing an increasingly important role in improving health outcomes and reducing health care costs. CQRs are established with the purpose of monitoring quality of care, providing feedback, benchmarking performance, describing pattern of treatment, reducing variation and as a tool for conducting research.

**Objectives:**

To synthesise the impact of clinical quality registries (CQRs) as an ‘intervention’ on (I) mortality/survival; (II) measures of outcome that reflect a process or outcome of health care; (III) health care utilisation; and (IV) healthcare-related costs.

**Methods:**

The following electronic databases were searched: MEDLINE, EMBASE, CENTRAL, CINAHL and Google Scholar. In addition, a review of the grey literature and a reference check of citations and reference lists within articles was undertaken to identify relevant studies in English covering the period January 1980 to December 2016. The PRISMA-P methodology, checklist and standard search strategy using pre-defined inclusion and exclusion criteria and structured data extraction tools were used. Data on study design and methods, participant characteristics attributes of included registries and impact of the registry on outcome measures and/or processes of care were extracted.

**Results:**

We identified 30102 abstracts from which 75 full text articles were assessed and finally 17 articles were selected for synthesis. Out of 17 studies, six focused on diabetes care, two on cardiac diseases, two on lung diseases and others on organ transplantations, rheumatoid arthritis, ulcer healing, surgical complications and kidney disease. The majority of studies were “before after” design (#11) followed by cohort design (#2), randomised controlled trial (#2), experimental non randomised study and one cross sectional comparison. The measures of impact of registries were multifarious and included change in processes of care, quality of care, treatment outcomes, adherence to guidelines and survival. Sixteen of 17 studies demonstrated positive findings in their outcomes after implementation of the registry.

**Conclusions:**

Despite the large number of published articles using data derived from CQRs, few have rigorously evaluated the impact of the registry as an intervention on improving health outcomes. Those that have evaluated this impact have mostly found a positive impact on healthcare processes and outcomes.

**Trial registration:**

PROSPERO CRD42015017319

## Introduction

Clinical registries play an important role in monitoring disease and healthcare delivery patterns and generating real world evidence of the impact of treatment and service delivery models on health outcomes [1–3]. Increasingly, clinical registries are being used in quality improvement projects to improve healthcare processes [4–11], adherence to clinical practice guidelines [9, 12–15] and standards [16–18]; and reduce the cost of delivering care [19–21]. Registries generally operate by providing details of clinical care, compliance with the evidence based guidelines and patient reported outcomes to participating hospitals [22–24] and to clinicians [1, 3, 25, 26].

Registries play an important role in medical research. Data within registries provide an ideal platform for randomized clinical trials, reducing both time and cost of prospective data collection [27]. The collection of real world data within registries supports research by generating research hypotheses [28–31], facilitating descriptive studies and health service research [29]. Research opportunities are enhanced when data from clinical registries are complemented with genomic, biomarker and imaging information [29, 32–35]. Registry data can be used to assist in answering research questions which are not practical or ethical to address by randomised controlled trials [36].

Gitt et al. [30] identified the following characteristics to be present in sophisticated clinical registries: standardized data collection methods and data definitions; integrated tools for rapid feedback to participating institutions/regulatory bodies; proper ethical review processes; electronic data capture; representativeness of the patient population under investigation; an audit process which assesses data accuracy; centralized data compilation and statistical analysis performed by professional statisticians; and appropriate and transparent reporting.

Health care systems costs in developed countries are rising, in part due to the introduction of advanced medical technology, pharmaceutical expenditure, hospital care and services as well as the high prevalence of chronic diseases and their financial consequence [37, 38]. One approach to address these challenges has been to invest in clinical quality registries (CQR). Sweden has established more than 100 national quality registries in the past decade [39]. A recent survey conducted by Lyu H et al [40] identified 153 national clinical registries in United States (US) of which 73 were health service registries, 66 were disease registries and 14 were combination of both health service and disease registries.

Examples of clinical quality registries can be found across most clinical specialities, monitoring both chronic and acute stages of illness. Acute stroke registries have been established in Sweden, Germany, Canada, Australia, the US, Argentina and many other countries [41–46]. The Cystic Fibrosis Foundation Patient registry has evolved from one built on an epidemiological and clinical research model to one which focuses also on fostering quality improvement processes [6]. The American Heart Association has a prolonged history of well-designed and successful implementation of different clinical registries and in their policy statement they highlight the importance expanding existing as well as developing new clinical registries [32].

Emilsson et al [39] recently reviewed 103 CQRs and concluded that Swedish quality registries contain comprehensive clinical data which complement information from government-administered registries and provide an important source for assessment and development of quality of care and research. CQRs help to identify variation in best practice treatments and in outcomes across treatment centres [47]. They provide feedback on performance, thereby encouraging quality improvement processes [48] and have been shown to identify factors that can impact survival across the countries [49, 50].

It is evident that the clinical registries, including CQRs, are being established as a tool to monitor and improve quality of care and as a platform for epidemiological research [51]. Yet, despite this, no systematic reviews have been undertaken to date to examine the impact of clinical registries as interventions on survival/mortality and on improving health outcomes. The objectives of this systematic review were therefore to understand the impact of CQRs on (I) mortality/ survival; (II) measures that reflects a process or outcome of health care; (III) healthcare utilisation; and (IV) healthcare-related costs.

## Methods

### Definition

A clinical registry is described as a system which collects a defined minimum data set from patients undergoing a particular procedure or therapy, diagnosed with a disease or using a health care resource [51].

In this systematic review we define CQR as a sub-group of clinical registries which systematically collect health-related information on the quality, safety and outcomes of care provided to individuals who are treated with a particular surgical procedure, device or drug; diagnosed with a particular illness; or managed via a specific healthcare resource [52]. CQRs are established primarily as tools for quality improvements and must provide feedback of data in reports to registry participants [51].

### Study design

Based on a pilot search it was evident that there were likely to be insufficient randomised controlled trial (RCT) studies assessing registries as an intervention to enable a meta-analysis to be undertaken. Therefore, a decision was made to include before-after, case control, cohort and controlled clinical trial study designs in addition to RCT studies.

A description of the population, intervention, comparison and outcome (PICO) [53] of the systematic review and a summary of the inclusion and exclusion criteria are described in “Table 1”.

**Table 1:** Description of the population, intervention, comparison and outcome (PICO) of the systematic review.

Sl#	PICO	Descriptions
1	Population	Studies conducted in clinical environments which may include acute care (inpatient and outpatient), sub-acute care (rehabilitation centre); AND community (general practice and aged care)
2	Intervention	Registry as an intervention with following inclusion and exclusion criteria. Inclusion criteria: Describe either a clinical registry or a CQR which collects data on a procedure, disease or healthcare resource; AND Collect data systematically and an ongoing basis from the population being investigated; AND Provide feedback on the performance of a health system on an ongoing basis; AND Collect data from more than one hospital. Exclusion criteria Studies will be excluded from the systematic review if they Collect and report on data from only one hospital; Do not provide feedback on an ongoing basis (such as an audit or point prevalence study); Are written in a language other than English; Were published prior to start date on January 01, 1980 or after the end date of December 31, 2016 or were review articles.
3	Comparison	Comparators: Data collecting tools other than registry (population based data, administrative data and medical record) to monitor health outcomes Studies without a comparator will be included
4	Outcome	Primary outcome measure is impact on survival/Mortality. Secondary outcome measures that reflects a process or outcome of health care, health care utilization and costs.

### Comparator(s)/Control

Studies with and without comparators were eligible for inclusion. Comparators were considered contemporaneous data sources such as hospital administrative databases, insurance databases and clinical information systems measuring mortality/survival; processes of care; outcomes other than mortality/survival; healthcare utilisation and costs.

### Context

Studies conducted in clinical environments which were considered acute care (inpatient and outpatient), sub-acute care (rehabilitation centre) and community (general practice and aged care) were included.

### Outcome measures/Outcome of interest

The primary outcome measure was impact of clinical registries, including CQRs, on survival or mortality. Secondary outcome measures were those reflecting a process or outcome of health care (quality of care), health care utilization and costs. Quality of care measures included those assessing impact of the registry on patient safety (complications), timeliness (reduce waits and harmful delays), effectiveness (adherence to clinical guidelines-provide services based on scientific knowledge), efficiency (avoid waste), equitable access to the services (provide services that does not vary in quality), and patient centeredness (provide care that is respectful of and responsive to individual patient preference, needs and values) [2].

### Search strategy

Before finalizing the search strategy, a pilot search strategy was developed, tested and amended as necessary across the different databases. Once the search strategy was finalized and agreed upon among the team, it was then refined by a senior medical librarian (LR). A database record was maintained at each stage of the review process including the detailing of how the search was undertaken and results of the search strategy.

The following electronic databases were searched: MEDLINE, EMBASE, Cochrane Central Register of Controlled Trials (CENTRAL), CINAHL and Google Scholar to identify studies in English covering the period January 1980 to December 2016. The search strategy included keywords describing studies involving established registries as the intervention. The MeSH terms relating to registry or registries combined with the MeSH terms referring to mortality, morbidity, patient reported outcome, health care utilization, cost/economic evaluation and clinical competence were included in the search.

Additional searches were conducted of grey literature resources such as conference websites and government websites, searching without limiting by outcome, searching informal sources such as conference abstracts and PhD theses. Hand-searching and reference checking of citations and reference lists were also undertaken.

### Study screening and selection

Titles and/or abstracts of studies identified using the search strategy and those from additional sources were distributed among four review authors. Two review team members independently retrieved and assessed the eligibility of the full text articles of potentially suitable studies. Any disagreement between the two reviewers was resolved through discussion with the third review author of the study team.

### Data extraction and assessment of quality of the study

A standardised data extraction form was developed and piloted, based on the template of the Cochrane data abstraction form [54]. Data extraction forms were used to extract data on study design and methods; country setting; participant characteristics; intervention characteristics including the feedback provided; study outcomes; discussion points; limitations; recommendations and study funding sources. Attributes of included registries were recorded using the criteria described by Black et al [55], and the adapted Scottish Intercollegiate Guidelines Network (SIGN) check list [53] was used to evaluate the methodology of included studies.

The first author (DEH) extracted the data; and inconsistencies were discussed with the second review author (VK) and if necessary with the third author (SE).

Our study protocol has been registered on PROSPERO: the international prospective register of systematic reviews (ID: CRD42015017319) and the study protocol has been described elsewhere [56].

### Data synthesis and analysis

#### Narrative synthesis

We followed the guideline for narrative synthesis for systematic reviews proposed by Popay J et al [57] as appropriate and developed a conceptual framework for analysis of registry outcome measures [56]. We conducted narrative synthesis of the findings from the included studies with special focus on the attributes of registries described as the intervention. Attributes included the host organization operating the registry; the reference population; coverage (extent to which the eligible population was representative of the country); personnel involved in the management of register; how data were being collected (paper based or electronic); the source of data and linkage with other database; availability of a data dictionary; the quality assurance procedure; and involvement of professional organizations and industry. We also conducted synthesis of the findings based on study design and impact of the registry on mortality/survival, process, utilization and clinical outcome measures.

#### Statistical analysis

Narrative synthesis of the selected articles was undertaken. It was not possible to conduct a meta-analysis because of heterogeneity among the study populations; coverage, duration and outcomes of interest, made it not possible to pool results. There was also variation among registries in follow-up, reporting mechanism, data management, quality assurance, audit mechanisms and processes; and feedback to stakeholders. Moreover, only two studies were RCT.

## Results

**Fig 1:**
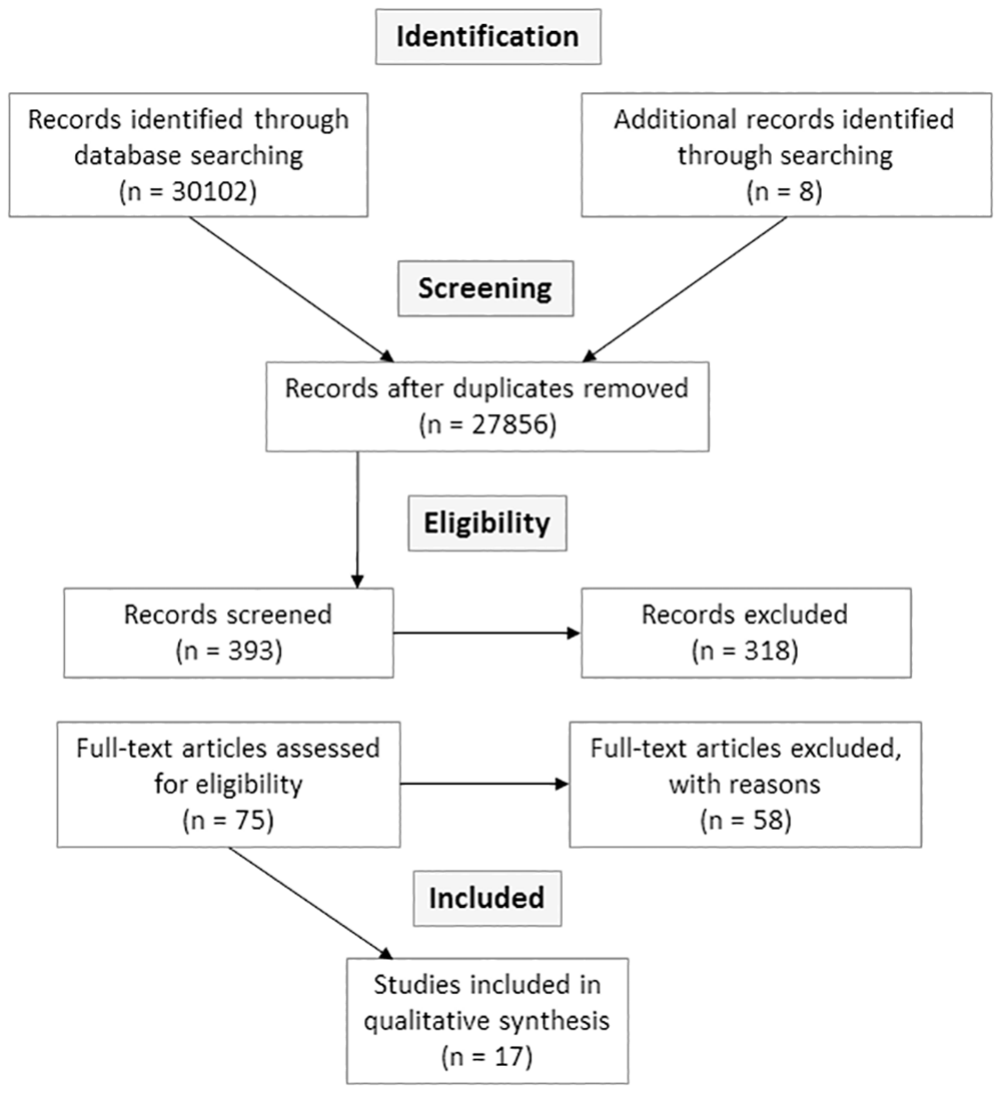
Results of search strategy following PRISMA flow diagram.

The results of our search strategy and selection process are presented in “Fig 1”, following the Preferred Reporting Items for Systematic Reviews and Meta-Analyses (PRISMA) flow diagram [58].

Through primary database screening, we identified 30102 articles. In addition, through hand searching, and reference searching of citations we identified an additional eight documents. After removal of duplications the numbers of abstracts reduced to 27856. Initially, 393 articles were screened after reviewing titles and abstracts. After reviewing 75 full text articles, a total of 17 articles were selected for final synthesis. All the selected journals were published between 2003 and 2015.

Details of the included studies and their Key characteristics are described in Table 2. Studies describe registries which were established in the US (n = 6), Australia (n = 2), the United Kingdom (n = 3), Denmark (n = 2), Sweden (n = 1), Germany (n = 1) and Israel (n = 1). Six studies described registries monitoring diabetes, two assessed stroke care, one each described care of patients with Chronic Obstructive Pulmonary Disease, lung cancer, colon cancer, rheumatoid arthritis, hard-to-heal ulcers, surgical complications, organ transplantation, Chronic Kidney Diseases; and one registry monitored patients undergoing smoking cessation therapy.

**Table 2:** Studies describing key characteristics of the registry as an intervention.

Reference	Condition of interest	Country where study conducted	Reference population	Coverage	Sources of data	Data base management personnel	Follow-up of registry participants	Reporting mechanism	Frequency of reporting	Nature of reporting	To whom Feedback provided
**RCT (#2)**											
Thomas KG et al, 2007 [59]	Diabetes	USA	Diabetic patients	484 patients of 78 IM residents in US.	Diabetic registry	Physicians	At 6 and 12- months	Registry generated feedback, list of patients and letter to patients	Quarterly	Automated, written report	IM resident and patients
Roski J et al, 2003 [60]	Smoking cessation	USA	Primary Care Clinics	40 clinics in the US	A centralized smoker registry and intervention registry	Service providers, counsellors at the clinics and trained medical record abstractor	Not stated Telephone survey, 6 months and 12 months	Not stated	Not stated	Not stated	Not stated
**Experimental non-randomised (#1)**											
Shah et al, 2015[61]	Diabetes	USA	Diabetes patients	All diabetic patients having a primary care visit and at least 1 recorded HbA1c lab value at 1 of 22 primary clinics	Electronic medical record based diabetes registry	Clinic administrator or personnel in charge of maintaining the registry	Yes, generation of reminder letters for the patients	Not stated	Not stated	Not stated	Not stated
**Prospective Cohort (#2)**											
Geubbels EL et al, 2006[62]	Surgical site infection (SSI)	Netherlands	Patients undergoing surgery	21,920 patients in 37 hospitals.	PREZIES^1^ or SSI and risk factors	Infection control personnel (ICP)	Not stated	An ICP^2^ responsible for quality of data collection and dissemination of results	Not stated	Dissemination and workshop	Infection control committee, Physicians, Mangers and staff
Tøttenborg SS et al, 2013 [63]	COPD^3^	Denmark	COPD patients aged ≥ 30 years	32018 patients presenting to OPC^4^ in Denmark.	DrCOPD ^5^	Doctors and care staff / nurses	Yes, but frequency not stated.	Not stated	Not stated	Not stated	Not stated
**Before After (#11)**											
Harris MF et al, 2006 [64]	Diabetes	Australia	Diabetic patients aged >25 years	16 divisions of general practice	Electronic diabetes patient register CARDIAB	GP^6^	None	Not stated	Not stated	Electronic	Not mentioned
Pollard C et al, 2009 [65]	Diabetes	USA	Diabetic patients	FQHCs^7^ in west Virginia.	CDEMS^8^	Lab company, Health care provider, Health centre personnel	After one year of onset implementation and then annually	Population and patient- based report, health centre based progress report and progress notes.	Quarterly	Electronic	An identified clinical champion in the clinic to facilitate the use of the report.
Goldfracht M et al, 2011 [66]	Diabetes	Israel	Diabetic patients	4 million patients enrolled in 2 HMOs covering 53% of the Israeli population	Computer based registry at clinic level and national level	Consultants, Primary Care Providers and Nurses	Yes but frequency not mentioned	Automated reminder, displaying outcomes, compound score	Quarterly	Web-based	Health care provider, Participating clinics and districts
Öien RF et al, 2013 [67]	Hard to heal Ulcer	Sweden	Patients with hard to heal ulcer	1417 patients in Sweden.	Registry of Ulcer Treatment	GPs and Nurses	Yes but frequency not stated	Feedback from online registry regarding quality of wound management	Real time	Electronic	Not stated
Hills NK et al, 2006 [68]	Stroke	USA	Transient ischemic attack (TIA) or ischemic stroke patients	86 hospitals in the US,	Ethos registry	Hospital staff	Not stated	Online access into the registry and aggregated report followed by self-feedback and performance improvement	Real time	Web-based	Hospital staff
Grau AJ et al, 2010 [41]	Stroke	Germany	TIA or stroke patients aged > 16 years old	70 hospitals with stroke unit of the Rhineland-Palatinate state of Germany.	Formal interview	Physicians	Yes but frequency not stated.	Detailed benchmark reports	6 monthly reports and annual meetings	Electronic	All participating hospitals
Young A et al, 2010[69]	Rheumatoid arthritis	England, Wales, Scotland and Ireland	Rheumatic disease patients	2866 patients managed in 30 rheumatology centres in England, Scotland and Ireland.	ERAN^9^ database	Rheumatologists or nurse	3–6 months, 12 months and then annually	Feedback of clinical practice and short-term outcomes	Rapid feedback of clinical practice and short-term outcome through Annual report.	Annual report, Annual general meetings, education trainings, publications	Participating centres
Salim A et al, 2010 [70]	Organ donation	USA	Organ donor	14 transplant centres and 220 hospitals in Southern California, US	The database from the Southern California regional OPO^10^	Not stated	Not stated	Not stated	Annually	Not stated	Participating clinics
Jakobsen E et al, 2013 [71]	Lung Cancer	Denmark	Lung cancer patients	All patient diagnosed with primary lung cancer are reported to DLCR from January 01, 2003 to December 31, 2012 in Denmark.	DLCR^11^	Representatives from personnel working in related sector, Multi-disciplinary group and national health authority	Yes but frequency not stated.	Reports from DLCG^12^/DLCR indicators results including descriptive analysis	Annual report and quarterly report. Adhoc reports with focus on specific issues like regional differences in treatment and survival.	Report published in a printed and a digital version.	All participating department, hospitals, regions, national level and an adjusted report to the public.
Mallinson E et al, 2010[72]	Colon Cancer	UK	FAP^13^ patients	Patients living within Genetic Registry and NWCIS^14^, boundaries which serve 4.7 million UK population.	Manchester Polyposis registry	Not stated	Yes, regular screening and post-surgical follow up but frequency not stated. Annual contact maintained	Not stated	Not stated	Not stated	Not stated
Harrison JK et al, 2015 [73]	Chronic Kidney disease	UK	Patients with advanced Chronic Kidney Disease (CKD)	Patients with CKD in four renal centres across the UK East Midlands	Electronic patient record based supportive care register	Any health care professional with access to the system primarily hospital physicians and specialist nurses	None	Not stated	Not stated	Not stated	Not stated
**Cross sectional (#1)**											
Harris MF et al, 2002 [74]	Diabetes	Australia	Pts of Diabetes Register user- and non-user- GPs.	614 GPs and 793 000 patients in South Australia, Australia.	AHI^15^ data base	GP	Every 6 months	Audit report; monthly list of overdue for GP to assist in patient recall.	6 monthly audit report; Monthly Recall Report	Electronic	GP

^1^ The Network for Prevention of Nosocomial Infections through Surveillance

^2^ Infection Control Personnel

^3^ Chronic Obstructive Pulmonary Disease

^4^ Outpatient Cinics

^5^ The Danish Clinical register of COPD

^6^ General practitioners

^7^ Federally Qualified Health Centers

^8^ Chronic Disease Electronic Management System

^9^ Early RA Network

^10^ Organ Procurement Organizations

^11^ The Danish Lung Cancer Registry

^12^ The Danish Lung Cancer Group

^13^ Familial Adenomatous Polyposis

^14^ North West Cancer Intelligence Service

^15^ Australian Health Insurance Commission

Table 3 describes the study design and impact of the registry on the primary and secondary outcomes and supplementary S1 Table summarises the limitations and conclusions/recommendations of the selected studies. Most used a “before-after” study design (n = 11, 65%) followed by RCT (n = 2, 12%) prospective cohort (n = 2, 12%), experimental non-randomised (n = 1, 6%) and cross sectional survey (n = 1, 6%). Each study except one study [61] assessing registries as interventions recorded some positive findings; however not all indicators of quality of care collected by the registry were positive.

**Table 3:** Study design, impact of registries on mortality/survival, process, utilization and clinical outcome measures.

References	Time period	Impact on mortality / Survival (Primary Outcomes)	Secondary outcome		
			Registry impact on processes of care	Registry impact on health service use	Registry impact on clinical outcomes
**RCT (#2)**					
Thomas KG et al, 2007[59]	2003–2004	Not measured	Significant improvement in intervention group compared to control group HbA1c testing in initial 6 months (61.5% vs 48.1%; p = .01) and LDL cholesterol testing in 12 months (75.8% vs 64.1%; p = .02).	Not reported	No significant impact were observed in intermediate clinical outcomes including HbA1c, LDL and blood pressure.
Roski J et al, 2003 [60]	1999–2000	Not measured	Significant improvement in Tobacco use identification improved by 14.4% in the incentive and 8.1% in registry group over control condition (6.2%). Patients visiting registry clinics accessed counselling programmes significantly (p<0.001) than patients receiving care in the control condition.	Not reported	Not reported
**Experimental non-randomised (#1)**					
Shah NR et al, 2015[61]	2010–2011	Not measured.	Diabetic patients receiving care at a clinic with a diabetes registry did not have significant impact on lowering HbA1c values than diabetic patients in clinics without the diabetes registry.	Not reported.	Not reported
**Prospective Cohort (#2)**					
Geubbels EL et al, 2006 [62]	1996–2000	Not measured.	Not reported.	Not reported.	Improvement in outcome (surgical site infection rate) over four years SSI stable at 4.3% in initial 3 years then reduced to 3.3% in year 4 and to 1.8% in year 5. The adjusted risk of SSI in 4th year of surveillance was reduced by 31% (95% Confidence Interval (CI) = 11% to 46%), and further decreased by 57% (95% CI = 24% to 76%) for 5th surveillance year.
Tøttenborg SS et al, 2013 [63]	2008–2011	Not measured.	Registration fulfilment increased more than 85% for all indicators. A higher proportion of COPD outpatients in 2011 received annual measurements of the FEV1 in 1 second [relative risk (RR) 2.14], BMI [RR 2.24], dyspnoea using the Medical Research Council scale [RR 2.25], registration of smoking status [RR 2.41], smoking cessation recommendation [RR 3.40] and offering of pulmonary rehabilitation [RR 2.78] compared to 2008.	Not reported.	Not reported.
**Before After (#11)**					
Harris MF et al, 2006 [64]	2000–2002	Not measured.	Significant improvement in processes of care: HbA1c, Systolic Blood Pressure, Diastolic Blood Pressure, Lipid levels in period 2002 compared to 2000. No significant changes in processes of care HDL, BMI, or frequency of renal and eye complications.	Not reported	Not reported
Pollard C et al, 2009[65]	2003–2006	Not measured.	Significant improvement from baseline on 12 of 13 care processes including foot, eye and dental examination, screening for depression, smoking, substance abuse when registry was used at a moderate level or higher; completed laboratory assessments for HbA1c and LDL at follow up and percentage of patients meeting American Diabetic Association (ADA) recommendations for LDL.	Not reported	Not reported
Goldfracht M et al, 2011 [66]	1995–2007	Not measured.	Significant improvement from baseline (1995) to 2007 in the intervention HMO^1^: HbA1c measurement: 22% (1995) vs 88% (2007). LDL measurement: 23% (1995) vs 89% (2007) Micro-albumin measurement: 10% (1995) vs 69% (2007).	Not reported.	Good control of LDL < 100 mg/dl increased from 26% to 59%.
Öien RF et al, 2013[67]	2009–2012	Not measured.	Not reported	Antibiotic treatment was reduced from 71% to 29% (p = 0.001).	Significant improvement from baseline in median healing time for all ulcers (146 days (2009) vs 63 days (2012), p = 0.001) and venous ulcer vs (120 days (2009) vs 69 days (2012, p = 0.001).
Hills NK et al, 2006 [68]	1999–2003	Not measured.	Not reported.	Longer time spent in registry was associated with 20% increased rates of in hospital antithrombotic use. Time in registry significantly associated with rates of treatment even when adjusted for calendar year (p = 0.0005).	Not reported.
Grau AJ et al, 2010 [41]	2001–2006	Hospital mortality ranged between 5.6% and 6.6% for ischemic stroke and between 14% and 20% for intracerebral haemorrhages with no temporal trends.	Number of patients registered: 6389 (2001) vs 10610 (2006) Admission < 3 hours after stroke onset: 28.2%(2001) vs 34.6% (2006). Admission via emergency systems: 38.2%(2001) vs 50.3% (2006). Diagnosis and treatment of hypertension and hyperlipidaemia, use of aspirin and combined aspirin/dipyridamole and diagnosis of atrial fibrillation increases (P<0.0001 respectively).	Use of thrombolytic therapy increased: 6.5%(2001) vs 14.1% (2006) Use of high-dose heparin declined: 24.5%(2001) vs 6.0% (2006) (P<0.0001). Referral by GPs declined (P<0.0001).	Not reported.
Young A et al, 2010[69]	1986–2002	Death from CVD^2^ commonest cause in the first 7 years of RA. Number of deaths from RA-ILD^3^ was 6%. The median survival following diagnosis of RA-ILD was 3 years.	A favourable change in time to first DMRD^4^ therapy and also an increase use of steroid before secondary care involvement.	Not reported.	An annualized incidence of RA-ILD was 4.1/1000 (95% CI 3.0, 5.4). 15-year cumulative incidence was 62.9/1000 (95% CI 43.0, 91.7).
Salim A et al, 2010[70]	2004–2008	Not measured.	Trends in consent rate (improved from 48% to 51.0% p = 0.064), conversion rate (improved from 45.0% to 50% p = 0.011), family decline rate (decreased from 44% to 33%, p<0.0001) and coroner decline rate (decreased from 1.8% to 0.6%, p = 0.004) improved in the post time.	There were 6,112 referrals during the pre-time period and 7,119 during post-time period. Extended criteria donors improved to 9.5% from 3.8% (p< 0.0001). Donor after cardiac death improved to 3.0% from 1.4% (p = 0.002). A decrease in organs per donor was noted (3.57% vs. 3.14%, (p< 0.0001).	Not report
Jakobsen E et al, 2013[71]	2003–2012	The 1-year overall survival rate: 36.6% (2003) & 42.7% (2011). 2-year survival rate: 19.8% (2003) & 24.3% (2010). 5-year survival rate: 9.8% (2003) & 12.1% (2007).	The rate of patients starting chemotherapy within 42 days after referral has improved 62.9% in 2003 to 82.9% in 2012, rate of patients with accordance between cTNM (clinical Tumour Nodes Metastasis) and pTNM (pathological Tumour Nodes Metastasis) was 68.2% in 2003 and 91.3% in 2012.	Not reported.	Not reported
Mallinson E et al, 2010 [72]	1969–2009	Survival was increased from 58.1 years to 69.6 year (p = 0.007) following establishment of Polyposis registry. Survival was increased from 57.8 years to 70.4 years (p<0.001) by screening.	Not reported.	Not reported.	The incidence of Colorectal Cancer reduced from 43.5% to 3.8% by screening and from 28.7% to 14.0% following establishment of Polyposis registry.
Harrison JK et al, 2015[73]	2009–2011	Not measured.	There was a 25.4% (95% CI: 6.5–44.3%, p = 0.008) improvement in patients having a documented discussion about end-of-life planning. There was also a 19.7% (95% CI: 4.0–35.5%, p = 0.01) improvement in establishing the place of death. Prior to the intervention only two patients (4%) were on the Gold Standards Framework Register at time of death. After the intervention 11 patients (27.5%) were registered, representing a 23.6% improvement (95% CI: 8.8–38.4%, p = 0.002).	After the intervention there was evidence of patients receiving input from a wider range of health professionals including social services and clinical psychology (p < 0.01). Palliative nursing care involvement was low pre-intervention with only one (2%) patient receiving this support, rising to 9 (22.5%) after the intervention, a 20.5% improvement (95% CI: 7.1–34.0%, p = 0.003).	Not reported
**Cross sectional (#1)**					
Harris MF et al, 2002[74]	1996–1998	Not measured.	Patients of register GPs were more likely to have more than one HbA1c test ordered within a six-month period than patient of non-register GPs for the first two years of the study period. There were also more likely to have at least one microalbuminuria test performed in each of the six-month period.	Higher number of patients and more likely to conduct tests based on recommended evidence based guidelines in registry users compare to non-users.	Not reported.

^1^ Health Maintenance Organization

^2^ Cardiovascular Diseases

^3^ RA-associated interstitial lung disease

^4^ Disease-modifying anti-rheumatic drugs

### Impact of the registry on mortality/survival

Four studies measured the registries association with either mortality or survival as the primary outcome [41, 69, 71, 72]; The registries established for lung cancer and colon cancer both demonstrated an association with improved survival after their development [71, 72]. Contrary to these findings, the registry established to monitor acute stroke care in Germany did not demonstrate any temporal trend on mortality [41]. Another study evaluated temporal trends in survival in patients diagnosed with rheumatoid arthritis (RA) [69]. The authors found that survival was significantly lower in the first seven years of RA than that was expected. The authors also found in the same population an excess mortality from cardiovascular diseases (31%) [75] and median survival time from RA-ILD was three years, with a five-year Kaplan-Meier survival function following diagnosis [76].

### Impact of the registry on secondary outcomes

#### (I) Impact on processes of care

With regard to the impact of the registry on process of care measures such as compliance with evidence-based guidelines, the findings were principally positive. The RCT by Thomas et al [59] assessing compliance with diabetes care using a registry versus normal care in internal medicine resident community clinics found that patients in the intervention arm were more likely to have HbA1c and LDL measured within the recommended time frame compared with those in the control arm (p<0.05). This did not, however, have a significant impact on overall control of HbA1c, LDL and blood pressure. The other RCT by Roski et al [60] investigating smoking cessation had three study arms; one provided financial incentives for reaching pre-set clinical performance targets (“incentive arm”); another provided financial incentives combined with access to a centralized smoker registry for reaching pre-set clinical performance targets (“registry arm”) while the third arm involved distribution of printed versions of the smoking cessation guidelines (“control arm”). The study demonstrated that the registry arm and the incentive arm were associated with greater documentation of tobacco use status (*P* < 0.01) than the control arm. Those patients visiting registry clinics reported accessing counselling programs significantly more often (*P <* 0.001) than patients receiving care in the control condition [60]. Jakobsen et al [71] demonstrated improvement in lung cancer survival rates, possibly due to improvement in the quality of care after introducing the registry.

Of six registries monitoring diabetes care assessed by HbA1c and lipid test results showed a positive benefit [59, 64–66, 74] of which two registries demonstrated positive impact on measurement of microalbuminuria [66, 74]. One registry monitoring diabetic care assessed by HbA1c did not find significant effects in lowering HbA1c values [61] and the study by Harris et al [64] did not show any significant change in processes of care for High- Density Lipoprotein (HDL), Body Mass Index (BMI), or frequency of renal and eye complications with registry use. A study by Pollard et al [65] reported significant improvements from baseline on 12 of 13 care processes indicators including foot, eye and dental examinations, and screening for depression, smoking, and substance abuse when a registry was used at a moderate level or higher. Diabetic registries including two in Australia and one in US [64, 65, 74], have clearly demonstrated a positive impact on patients enrolled in a registry compared to those who are not. This is thought to be due to the capacity of a registry to generate progress notes and summary reports to track patients’ care and monitor compliance with guidelines [65].

#### (II) Impact of healthcare utilization

One study reported that use of a diabetic registry was associated with a higher average number of patients and attendances by patients with diabetes per GP compared to those not using a registry [74]. Öien et al [67] demonstrated that following the introduction of a registry for ulcer treatment, treatment with antibiotics reduced significantly from 76% in 2009 to 24% in 2012 (p = 0.001). Other studies have shown an association between registry use and higher rates of uptake of evidence based practice, [41] and that impact on practice improves over time [68].

#### (III) Impact on clinical outcome

Öien et al found that there was a significant reduction of healing time for hard-to-heal ulcers when the registry was used 21 weeks in 2009 to 9 weeks in 2012. Geubbels et al [62], reported that surgical site infection was stable at 4.3% in initial 3 years then reduced to 3.3% in year 4 and to 1.8% in year 5. Good control of LDL<100mg dl increased from 26% to 59% has been reported by one registry [66].

#### (IV) Impact on healthcare costs

None of the 17 studies included in this review rigorously evaluated the cost effectiveness of the clinical registry.

One registry has demonstrated significantly reduced regional difference of improving clinical practice and core results [71].

## Discussion

In this review our primary objective was to establish the impact of registries, including CQRs, on mortality or survival. We identified four articles which reported on survival, of which two demonstrated a positive impact. Our secondary objectives were to assess impact on processes or outcome of health care other than survival or mortality, health care utilization, clinical outcome and cost. We identified 17 articles which assessed the impact of clinical registries on secondary outcomes of which all articles reported a positive impact except one [61] and few process indicators of two articles [59, 64]. Five out of six registries assessing diabetic care demonstrated at least one improvement in processes of care [59, 64–66, 74]. Most studies (n = 11) used a before-after study design [41, 64–73]; only two studies were experimental in design with one assessing the impact of a registry on diabetes management and the other on tobacco smoking. Both showed significant improvement in health outcomes after implementation of the registry [59, 60].

Our finding that few studies have rigorously evaluated the impact of clinical registries as interventions on improved health outcomes may be explained by a number of factors. Firstly, we found it difficult to disentangle registries as interventions from larger quality improvement projects which use registries to store data. For example, the Get with the Guidelines (GWTG) Stroke, Coronary Artery disease and Heart failure programs collect data on patients with these diseases and report performance measures back to hospitals as part of a quality improvement cycle [77, 78]. The Patient Management Tool (PMT) used in the GWTG program to collect data and provide decision support and reports back to hospitals might be considered a registry. However, it was only one element of the program, which also included organizational stakeholder and opinion leader meetings, hospital recruitment, collaborative learning sessions, hospital tool kits, local clinical champions, multidisciplinary teams, and hospital recognition. The program cannot disengage the effect of the PMT from other activities and, for this reason was not included in this review.

Another reason for the limited number of studies evaluating registries as interventions may be that researchers have not established them with quality improvement as an outcome in mind. Registries are often established to describe patterns of care, to understand variation in treatment and outcomes and predictors of prognosis and quality of life. Yet, many have not been set up to report quality of care data back to health services. Often population-based registries store data for reporting to government and for research purposes but fail to collect data which is meaningful to health services to assess their performance or, if they, do not make it available to the health services, so that they can see how they compare with others and with themselves over time.

Finally, it may be that there were few studies evaluating the impact of registries because researchers and sites investing heavily in the development of registries have been reluctant to formally assess their impact through rigorous trial design. Establishing a registry requires substantial resources, infrastructure and sustained funding [29].

While we found no studies during our study period of investigation to December 2016 comparing the cost effectiveness of registries, two articles have provided supportive evidence of cost-effectiveness of investing in the registry [20, 79] and a report has recently been released assessing the cost-effectiveness of five Australian CQRs [80]. Larsson et al [20] presented a case study for estimating the cost saving of a hip registry by calculating direct medical savings through reduction in complications and avoided revision operations. By comparing hip revision rates in the US against those achieved in Sweden, where a registry exists with a revision rate of 10%, Larsson concluded that the US could potentially save $2 billion or 8% of the total costs of an expected $24 billion in total costs for these surgeries in 2015. Another study extrapolated the costs of emergency care for diabetics [81] and reported that a registry for quality improvement of type-2 diabetics in eight Western New York counties might lead to an estimated annual savings of more than $700 million through a 20% reduction in emergency department visits [79]. The Australian Commission on Safety and Quality in Health Care study assessing the economic impact of five Australian CQRs, and concluded that they provided a significant net positive return on investment, with benefit to cost ratios ranging from 2:1 to 7:1 [80].

This is the first review which has rigorously evaluated the impact of registries as interventions on quality of care. We found that registries play an important role in care management processes through generating performance feedback reports to physicians [59, 66], helping to identify patients who are not receiving treatment in accordance with guidelines [69, 74], creating a trigger for action by physicians [65, 74], creating a reminder for patients [59, 64], identifying high-risk patients so they can be more closely monitored [82] and reducing regional differences [71]. Registries have provided demonstrable improvement in processes of care and clinical outcomes for patients with chronic diseases [73, 83, 84]. In our review five different studies demonstrated that use of registries as tools for physician’s reminder, patient recall, structured care, audit, and progress notes can improve documentation which, in turn, leads to improvement in diabetic care [59, 64–66, 74].

Our review has several limitations. These include the quality of the studies and the heterogeneity of the study populations, coverage, duration and outcomes of interest, making it impossible to pool results. We found variation in follow-up, reporting mechanism, data management and quality assurance, and audit of data within the registry. Reporting and feedback mechanisms of the registries to stakeholder were also diverse; an issue similar to other review findings [85].

Only two [59, 60] of the 17 studies used a randomised controlled clinical trial design. Both were adequately powered to assess the impact of a registry design on improving quality of care, yet details of the intervention were poorly described in one of the studies, making it difficult to ascribe the success to the registry. Two-thirds of studies evaluating registries used the less robust before-and after- quasi-experimental study design. Limitations of this design were discussed by authors and include inability to attribute causality to the registry [59, 68]. While most studies commented that the registry likely accounted for improvement in outcomes and processes of care, they also acknowledged that the design of the studies made it impossible to clearly quantify this impact (Supplementary S1 Table).

While effort was made to minimise publication bias by including a broad search strategy including both the academic and grey literature, it may be that further evidence exists on the impact of registries on health outcomes, care utilization and costs but is not yet published. As we limited our search by English language, we may have missed relevant article which published in other languages. However, the major shift towards publication of studies in English, the extent and effects of language bias may have recently been reduced.

As registries continue to proliferate, there is a need to more rigorously evaluate their impact within and across disease conditions. This includes identifying what attributes of registries are most effective and which have little or no impact. Registries should consider using progressive study designs such as stepped wedge RCTS. This provides the advantage of allocating all sites the intervention at some point in time [86]. Because most registries initially start as a pilot e and expand over time, this provides a good opportunity to consider a step wedge design intervention to assess impact more rigorously. Moreover, in evaluating costs and outcomes attributable to clinical registries sufficient time should be provided to allow hospitals to set up appropriate governance structures, collect enough data to generate meaningful reports and a number of feedback cycles to enable hospitals and clinicians to act on findings.

## Conclusions

Despite the large number of published articles using data derived from clinical registries, few have rigorously evaluated the impact of the registry as an intervention on improving health outcomes; those that have evaluated this impact have mostly found that registries have improved healthcare processes and outcomes. No studies have evaluated the economic impact of registries as an intervention.

## Supporting information

S1 Table Study limitations, conclusions and recommendations. (DOCX)

S1 Appendix PRISMA 2009 checklist. (DOCX)
