# Supplementary material for: Impact of clinical registries on quality of patient care and clinical outcomes: A systematic review
Source: PLoS One. 2017 Sep 8;12(9):e0183667. doi: 10.1371/journal.pone.0183667 (PMC5591016; doi:10.1371/journal.pone.0183667)
Supplement: S1 Table — (DOCX) [file pone.0183667.s001.docx]

**S1 Table: Study limitations, conclusions and recommendations.**

| **References** | **Limitations** | **Conclusions/Recommendations** |
| --- | --- | --- |
| **RCT** | | |
| Thomas KG et al, 2007 [59] | - Resident participation in the intervention was incomplete. - 59.1 % of resident completed two diabetic sessions on diabetic registry use. - The study was unable to quantify differential effects of intervention components. - Blinding was not possible because of the nature of the intervention. | Integration of registry-generated audit, feedback and patient reminders in a registry improves adherence to diabetes care process which indicates registry could be useful tools in managing diabetic care as well as chronic diseases. |
| Roski J et al, 2003 [60] | - Not stated. | In this study for improving smoking cessation clinical practices and patient behaviours in the impact of financial incentives and a patient register was mixed. Further research is needed to determine under what conditions the potentially costly implementation of financial incentives or patient registries with coupled intervention system provide sufficient gains in health care quality and to justify the investment. |
| **Experimental non-randomised** | | |
| Shah NR et al, 2015 [61] | - HbA1c were collected for 2-year time period; greater differences over time may have been observed between diabetes registry/non-diabetes registries if a longer study period was studied. - Missing information where fewer than 3 HbA1c values were available which seems to underestimate any observed differences. - Time between patient visits or collection of HbA1c values were not consistent across groups. - Prevalent, rather than incident disease status was measured; this is important since the trajectory for reducing HbA1c values may be different for patients newly diagnosed with diabetes compared to those who have been managing their diseases for some time. | More careful evaluation is required to determine whether registries are effective tools to reduce diabetes morbidity and mortality. |
| **Prospective Cohort** | | |
| Geubbels EL et al, 2006 [62] | - Selection bias might have occurred as not all the hospitals that participated in PREZIES took part in this study. | Hospitals can reduce their Surgical Site of Infection (SSI) rates by enrolling in a surveillance network. It is a tool for continuous quality improvement and can provide feedback about the adverse health outcome and enable them to take appropriate action. |
| Tøttenborg SS et al, 2013 [63] | - The limitations of this study include a high proportion of missing data for some of the variables in early years. - A number of the COPD patients who were misdiagnosed as having asthma may not be included in the register. | Substantial improvement in the quality of care of COPD patient in Danish hospitals has increased due to registration practices of important process of care together with initiation of multi-disciplinary quality improvement program. |
| **Before After** | | |
| Harris MF et al, 2006 [64] | - Patients on the diabetes register represented only 10% of those estimated to have diabetes in 2002. - This was an observational study, so causality of improvement was not ascertained. | Registry provide useful data in managing diabetes care in primary health care setting and improve process of care and clinical outcomes. |
| Pollard C et al, 2009 [65] | - Use of a non-randomly assigned control group and the inclusion criterion that required patients to have both baseline and follow-up care. - It is not clear from the results of this study if the observed improvement in care that corresponded to registry utilization are improvements initiated by the registry or improved documentation. | Basic registry utilization may be sufficient to improve provider-patient care processes and patient outcomes in rural clinics with minimum resources. |
| Goldfracht M et al, 2011 [66] | - The study design was an observational quasi-experimental design and not a clinical trial, so it has some intrinsic limitations. - One important limitation was absence of a control group within Clalit’s, due to Clalit’s policy of including all of its population in quality improvement programs. - Reliability of Clalit’s data were low during 1995-1999 because of manual collection. - The lack of consistency in the data systems over time may have also introduced bias into the findings. - The available data did not allow a hierarchical analysis dealing with the potential nesting and clustering effects at the practice and clinic level. - It was not possible to assess the association between the levels of practice participation in various aspects of the level of improvement of outcomes. | The quality of diabetic care patients could be improved significantly if centre registry is being implemented together with multi-faceted interventions like infrastructure changes, guidelines, Continuous Medical Education (CME) session focused on follow-up, feedback, reminders and tools for patient empowerment. |
| Öien RF et al, 2013 [67] | - Generalisability to region outside where the registry was developed is unclear. A large number of the patient population 39% of the registry was concentred in the Blekinge Country which may lead to bias because the registry was developed in this County before being expanded to the whole country. | The use of national quality registry for structured ulcer care demonstrated as an improvement project within wound management with significant reduction of healing time and the potential for improved management of wound. |
| Hills NK et al, 2006 [68] | - Unable to adjust for other hospital-level interventions that might have contributed in the improvement process. As other studies [87, 88] have shown that to produce substantial impact from registry data individualized feedback is necessary, so it was impossible to prove the solely attribute of registry participation in improvement of care as opposed to other simultaneous interventions. - As the hospitals participation in the registry was voluntary in nature there were likely to be participated those who were interested in improving the quality of stroke care, thus the results may have been influenced by selection bias. - Entry of Selective patients into the registry potentially could have biased the study results. - There were no control or comparison hospitals. | Although voluntary diseases registries have limitations due to sampling and limited supervision on data quality but they have been a major sources of important health services and outcomes research in other disease areas like Registry for Myocardial Infarction. Other voluntary registries in various disease areas have demonstrated the source of important research and basis for quality improvement process [73, 89, 90]. |
| Grau AJ et al, 2010 [41] | - Incomplete acquisition of data for patients treated in neurosurgical departments. - The possibility of duplication of registration by different hospitals. - Lack of any on-site data monitoring. - The questionnaire that aimed at simplicity of data acquisition by physicians does not allow differentiation between the absence of a diagnosis or procedure and missing information for several parameters. | The registry emphasizes the concept that monitoring of quality of stroke care is feasible on a state or country wide and helpful to improve and maintain in larger population. The most important and successful aspects of quality monitoring processes were increasing rates of using emergency medical services, arriving early in hospital and increasing use of thrombolytic therapy. |
| Young A et al, 2010 [69] | - Main source of missing data was for drug start and stop dates were around 10%. - Limited criteria are available for severity of comorbid conditions - Though the effect of the register on clinical practice have as yet not been made, indirect effects are apparent. | The RA registry provides insight into the natural history and impact of RA, its management, translation of research findings into clinical practice and provides participating centres clinical governance and professional development. Results from RA registry greatly enhanced by linkage with national database. |
| Salim A et al, 2010 [70] | - The retrospective design of the database limits the conclusions. - It was not possible to analyse the direct effect of the educational campaign on organ donation rates. - As the collaborative were present throughout the study period, it is possible that they had a role in the favourable results. - Even though the conversion rate improved significantly in the second time period, it was still far below the 75% rate established by the organ donation collaborative. | An organ donation registry together with public donation campaign have positive effect on increasing organ donation. However, despite significant increases in the consent and conversion rates, further work is required to address the organ shortage crisis and educational campaign should be considered as a method to influence organ donation. |
| Jakobsen E et al, 2013 [71] | - Not stated | The Danish DLCG and DLCR experience shows that a national comprehensive quality management system together with national guidelines, a database with high quality data and completeness, frequent reports to the professionals and public, audit and commitment from all stakeholders can contribute to improve practice and results and reduce regional differences. |
| Mallinson E et al, 2010 [72] | - Not stated | The implementation of both a Registry and screening has resulted in reduction in incidence of colorectal cancer. |
| Harrison JK et al, 2015 [73] | - The sample is relatively small and describes the experiences of the intervention in the study region. However, there was no evidence to suggest that they were not generalisable. | These results show that the interventions implemented to introduce a supportive care register resulted in meaningful improvements to the end-of-life care for patients in the study areas with advanced Chronic Kidney Diseases. |
| **Cross sectional** | | |
| Harris MF et al, 2002 [74] | - The possibility of characteristics of practices impacting quality of care was not assessed. - Improvement may be due to (I) improved awareness of the needs of diabetic patients through the registry; (II) selection bias- GPs with greater interest in quality of care are more like to enrol patients in a register or (III) characteristics of the practice like size, location and management practice may influence the quality of care and participation in the registry. | Registry play an important role in compliance with evidence based guideline for diabetic patient care by GPs. Further research requires to reveal whether this was the results of characteristics of the GPs themselves or their practices or was a consequence of their participation in the registry. |

87. Bradley EH, Holmboe ES, Mattera JA, Roumanis SA, Radford MJ, Krumholz HM. A qualitative study of increasing β-blocker use after myocardial infarction: why do some hospitals succeed? Jama. 2001;285(20):2604-11.

88. Bradley EH, Holmboe ES, Wang Y, Herrin J, Frederick PD, Mattera JA, et al. What are hospitals doing to increase beta-blocker use? The Joint Commission Journal on Quality and Patient Safety. 2003;29(8):409-15.

89. Fonarow GC, Abraham WT, Albert NM, Gattis WA, Gheorghiade M, Greenberg B, et al. Organized program to initiate lifesaving treatment in hospitalized patients with heart failure (OPTIMIZE-HF): rationale and design. American heart journal. 2004;148(1):43-51.

90. Bradley EH, Herrin J, Mattera JA, Holmboe ES, Wang Y, Frederick P, et al. Hospital-level performance improvement: beta-blocker use after acute myocardial infarction. Medical care. 2004;42(6):591-9.
